# A nomogram for predicting the risk of cerebral vasospasm after neurosurgical clipping in patients with aneurysmal subarachnoid hemorrhage

**DOI:** 10.3389/fneur.2024.1300930

**Published:** 2024-02-16

**Authors:** Yu Zhou, Dongliang Qian, Zhou Zhou, Bin Li, Yong Ma, Erqing Chai

**Affiliations:** ^1^First Clinical Medical College, Lanzhou University, Lanzhou, China; ^2^Cerebrovascular Disease Center, Gansu Provincial Hospital, Lanzhou, China; ^3^Key Laboratory of Cerebrovascular Diseases, Lanzhou, China; ^4^Clinical Medicine College, Ningxia Medical University, Yinchuan, China; ^5^First Teaching Hospital of Tianjin University of Traditional Chinese Medicine, Tianjing, China; ^6^National Clinical Research Center for Chinese Medicine Acquisition and Moxibustion, Tianjin University of Traditional Chinese Medicine, Tianjing, China

**Keywords:** intracranial aneurysm, subarachnoid hemorrhage, cerebral vasospasm, neurosurgical clipping, nomogram

## Abstract

**Purpose:**

Cerebral vasospasm (CVS) is a common complication that occurs after neurosurgical clipping of intracranial aneurysms in patients with aSAH. This complication can lead to clinical deterioration and a poor prognosis. The aim of this study is to explore the risk factors for CVS in aSAH patients who have undergone neurosurgical clipping, develop a nomogram for CVS, and evaluate its performance.

**Methods:**

Patients with aSAH who underwent neurosurgical clipping in the Department of Neurosurgery from January 2018 to January 2023 were selected as the subjects of this research. The clinical data of these patients were retrospectively analyzed. Logistic multivariate regression analysis was employed to identify the independent risk factors of CVS. A clinical prediction model in the form of a nomogram for CVS was developed using the R programming language and subsequently evaluated for its performance and quality.

**Results:**

A total of 156 patients with aSAH were included in the analysis, comprising 109 patients in the training set and 47 patients in the validation set. In the training cohort, 27 patients (24.77%) developed CVS after neurosurgical clipping, while in the validation cohort, 15 patients (31.91%) experienced CVS. Multivariate regression analysis revealed that age, Hcy, WBC, glucose/potassium ratio, aneurysm location, and modified Fisher grade were independent risk factors for CVS. The nomogram exhibited excellent discriminative performance in both the training set (AUC = 0.885) and the validation set (AUC = 0.906).

**Conclusion:**

CVS was a prevalent complication following neurosurgical clipping in patients with aSAH, with a highly intricate pathogenesis and pathophysiological course. Early prediction of CVS represented a significant challenge in clinical practice. In this study, age, Hcy, WBC, glucose/potassium ratio, aneurysm location, and modified Fisher grade emerged as independent risk factors for CVS. The resulting nomogram demonstrated substantial predictive value.

## Introduction

Spontaneous subarachnoid hemorrhage (SAH) constitutes a particularly perilous form of cerebrovascular ailment, characterized by its abrupt onset. Despite the rapid advancements in medical care, its mortality and disability rates remain notably elevated (1–6). Eighty percent of these spontaneous SAH cases result from the rupture of intracranial aneurysms (7, 8). As a classic neurosurgical operation for the treatment of aneurysmal subarachnoid hemorrhage (aSAH), neurosurgical clipping is predominantly conducted under direct visual guidance employing advanced tools such as microscopes and endoscopes, facilitated by the ongoing evolution of technology (9). This surgical technique not only affords complete visualization of the aneurysm’s location, size, and quantity under direct observation but also enables the prompt removal of subarachnoid hemorrhage resulting from aneurysm rupture during the procedure.

Cerebral vasospasm (CVS) stands as a prevalent complication following clipping procedures in patients afflicted by aSAH. Yalamanchili et al. (10) conducted a comparison among patients with the same Hunt-Hess and Fisher classifications who underwent craniotomy clipping and endovascular embolization within 48 h after subarachnoid hemorrhage. In the embolization group, 22% of patients experienced cerebral vasospasm, while in the craniotomy clipping group, the incidence rose to 74%. Therefore, heightened vigilance towards the potential occurrence of CVS is advisable for patients undergoing clipping surgery. Currently, even in highly established cerebrovascular disease centers, clinicians can only identify CVS based on a patient’s clinical deterioration and take a more aggressive anti-CVS treatment strategy in most cases. The diagnosis and treatment of CVS remain in a somewhat passive state. Once CVS occurs, it can yield severe consequences and markedly alter patients’ prognoses and quality of life. Hence, the early prediction of CVS assumes paramount importance in clinical practice. This study, through retrospective analysis of clinical cases, aimed to investigate the risk factors associated with CVS in patients with aSAH following neurosurgical clipping. Subsequently, it sought to construct a clinical prediction model in the form of a nomogram, with plans for its conversion into a practical clinical tool following a rigorous assessment of the model’s performance and quality.

## Materials and methods

### Patient population and research methods

This study was a single-center retrospective control study. Two hundred twenty-seven aSAH patients who were treated hospital from January 2018 to January 2023 were selected. This study was approved by the Clinical Research Ethics Committee of Hospital, and all methods were performed in accordance with the relevant guidelines and regulations. All patients were informed and agreed to participate in this study.

Inclusion criteria. (1) Spontaneous SAH caused by ruptured intracranial aneurysm, in which intracranial aneurysm was diagnosed by imaging examination [computed tomography angiography (CTA), digital subtraction angiography (DSA), etc.]. (2) Underwent neurosurgical clipping. (3) Complete admission clinical data.

Exclusion criteria. (1) Non-aneurysmal subarachnoid hemorrhage, including traumatic subarachnoid hemorrhage, subarachnoid hemorrhage with negative cerebral angiography, and subarachnoid hemorrhage caused by cerebral arteriovenous malformation, blood diseases, vasculitis, etc. (2) History of stroke, brain tumor, or severe head trauma. (3) With severe systemic disease such as heart failure, renal failure, uremia, etc. (4) Combined with other cerebrovascular malformations such as moyamoya disease. (5) Cases that were in a near-death state on admission (Hunt-Hess grade V), so that various surgical treatments could not be performed. (6) Incomplete admission clinical data.

**Figure 1:**
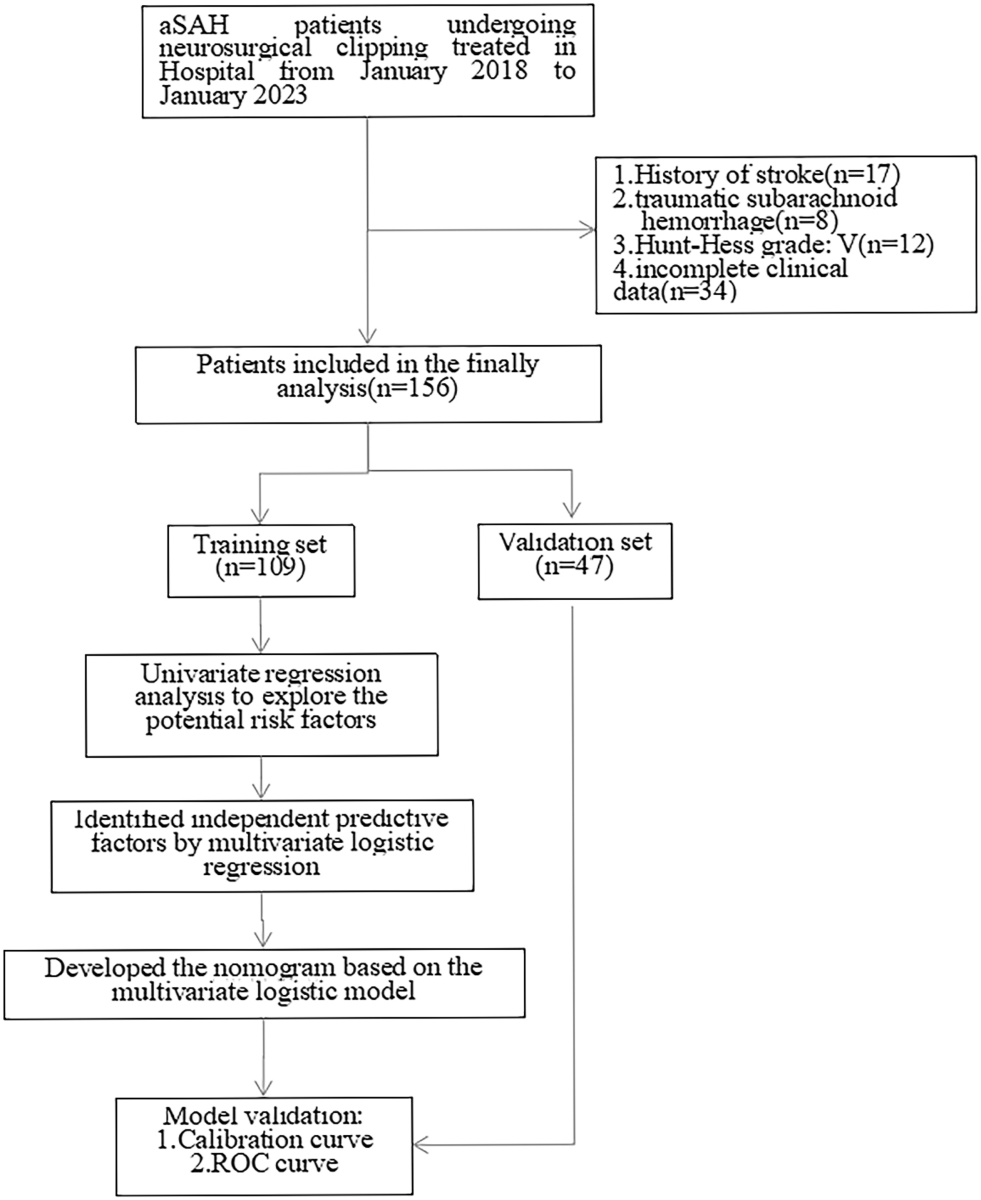
Schematic of patient’s inclusion process and flow chart with the study.

After exclusion of 17 patients with a history of stroke, 8 traumatic subarachnoid hemorrage patients, 12 Hunt-Hess V grade patients, 34 patients without complete data, 156 patients were included in the statistical analysis who were randomly divided into training set and validation set with a ratio of 70%:30% (Figure 1).

### Exposure variables

The case data were retrieved and sorted through the Hospital Electronic Medical Recording System and Hospital Information System platform, mainly including clinical characteristics and laboratory results; Subjective indicators were confirmed by two or more experienced clinicians/technicians. Objective indicators were obtained directly: (1) clinical characteristics: age, gender, Hunt-Hess grade (11), modified fisher grade (12), hypertension, and smoke; (2) aneurysm characteristics: aneurysm location (anterior circulation aneurysms or other location of the aneurysm), aneurysm size (small: ≤0.5 cm, general: 0.5 cm–1.5 cm; large: 1.5–2.5 cm; giant: ≥2.5 cm), aneurysm hemorrhages times: 2 < or ≥ 2; (3) laboratory tests (it is directly acquired by the HIS platform): admission blood Ca^2+^ level, homocysteine (Hcy), white blood cell count (WBC), platelets (PLT), hematocrit (HCT), red blood cell count (RBC) and hemoglobin (Hb), glucose/potassium ratio.

### Study group and diagnosis of CVS

The outcome variable was CVS after neurosurgical clipping; Patients were divided into CVS^+^ group if CVS occurred after neurosurgical clipping, otherwise they were divided into CVS-group.

At present, there was no clear and unified standard for the definition of CVS. Therefore, based on the literature and clinical experience, we define its diagnostic criteria as follows: (1) the symptoms of aSAH were improved and stable after treatment, but deteriorated or aggravated again; (2) fever and elevated white blood cell count were observed, but there was no definite signs of infection; (3) there were focal manifestations at the site of vasospasm, such as hemiplegia, aphasia and so on; (4) recurrent symptoms of suspected intracranial hypertension (headache, vomiting, papilledema), continuous, intense and difficult to relieve headaches; (5) definitive radiological evidence: DSA revealed CVS, or transcranial doppler indicated peak blood flow velocity in the middle cerebral artery >200 cm/s or mean blood flow velocity >120 cm/s, indicating CVS.

If any one or more items of 1–4 plus 5 were present, and acute hydrocephalus, distant intracranial hematoma, severe electrolyte disturbance, etc. were excluded, CVS could be basically diagnosed, which was confirmed by 2 neurosurgeons with associate senior titles or above.

### Statistical analysis

Statistical analysis was conducted using SPSS 26.0 and R software (version 4.3.0). Continuous variables following a normal distribution were presented as mean ± standard deviation, while non-normally distributed variables were described using the interquartile range. Categorical variables were expressed as frequency (percentage).

Univariate analysis was employed to identify potential risk factors for CVS in patients with aSAH following neurosurgical clipping. To ascertain independent risk factors for CVS, variables with *p* < 0.05 in the univariate analysis were included in a multivariate logistic regression model. Subsequently, a predictive nomogram was developed based on the independent risk factors using the ‘rms’ package in R software. Receiver operating characteristic (ROC) curves were generated, and the area under the curve (AUC) was used to assess the nomogram’s discriminatory power. Additionally, the AUC of the nomogram was compared to that of all the independent risk variables. Statistical significance was defined as *p* < 0.05 (two-sided tests), and regression coefficients were reported with 95% confidence intervals (CI).

## Result

### Clinical characteristics of patients in the training and validation set

A total of 156 patients with aSAH were included in the analysis, comprising 109 patients in the training set and 47 patients in the validation set. In the training cohort, 27 patients (24.77%) developed CVS after neurosurgical clipping, while in the validation cohort, 15 patients (31.91%) experienced CVS. There were no significant differences in patient characteristics between the training and validation sets, as indicated in Table 1 (*p* > 0.05).

**Table 1:** Baseline and clinical characteristics of patients in the training set and validation set.

Clinical data	Training set (*n* = 109)	Validation set (*n* = 47)	*t*/*z*/*χ*^2^	*p*-value
Man (%)	59 (54.13)	24 (51.06)	0.124	0.725
Age	50.79 ± 7.94	50.66 ± 7.90	0.093	0.926
Hypertension (%)	54 (49.54)	18 (38.30)	1.670	0.196
Smoke (%)	35 (32.11)	19 (40.43)	1.003	0.317
Subarachnoid hemorrhage times (%)			2.960	0.085
<2	88 (80.73)	32 (68.09)		
≥2	21 (19.27)	15 (31.91)		
Aneurysm size (%)			0.811	0.847
≤5 mm	61 (55.96)	23 (48.94)		
5–15 mm	38 (34.86)	19 (40.43)		
15–20 mm	7 (6.42)	3 (6.38)		
≥20 mm	3 (2.75)	2 (4.26)		
Aneurysm location (%)			1.412	0.235
Anterior circulation aneurysm	53 (48.62)	18 (38.30)		
Other	56 (51.38)	29 (61.70)		
Modified Fisher grade (%)			1.775	0.620
I	36 (33.03)	16 (34.04)		
II	50 (45.87)	17 (36.17)		
III	18 (16.51)	11 (23.40)		
IV	5 (4.59)	3 (6.38)		
Hunt-Hess grade (%)			7.468	0.058
I	18 (16.51)	14 (29.79)		
II	75 (68.81)	22 (46.81)		
III	11 (10.09)	9 (19.15)		
IV	5 (4.59)	2 (4.26)		
Ca^+^ (mmol/L)	2.50 (2.31, 2.71)	2.45 (2.16, 2.58)	−1.953	0.051
Hcy (μmol/L)	17.00 (12.50, 22.50)	15.60 (13.35, 19.20)	−0.465	0.642
PLT (10^9^/L)	209.67 ± 56.18	215.87 ± 47.76	−0.657	0.512
RBC (10^12^/L)	4.99 ± 0.52	4.80 ± 0.59	1.955	0.052
Hb (g/L)	144.18 ± 14.65	143.11 ± 12.63	0.436	0.664
HCT (%)	44.62 ± 3.47	44.73 ± 3.35	−0.173	0.863
WBC (10^9^/L)	8.8 (6.7, 13.6)	8.8 (7.4, 12.2)	−0.052	0.958
Glucose/potassium ratio	1.32 (1.24, 1.65)	1.31 (1.22, 1.60)	−0.844	0.399

Hcy, homocysteine; PLT, platelet; RBC, red blood cell; Hb, hemoglobin; HCT, hematocrit; WBC, white blood cell.

### Baseline characteristics of the patients in the two groups in the training set

Descriptive analysis showed that there were significant differences in age (*p* = 0.011), hypertension (*p* = 0.040), subarachnoid hemorrhage times (*p* = 0.033), aneurysm location (*p* = 0.009), modified Fisher grade (*p* = 0.006), Hunt-Hess grade (p = 0.011), Hcy (*p* = 0.002), Plt (*p* = 0.033), WBC (*p* = 0.021) and glucose/potassium ratio (*p* = 0.001) between the two groups (Table 2).

**Table 2:** Baseline and clinical characteristics of patients in the training set.

Clinical data	CVS^+^ (*n* = 27)	CVS^−^ (*n* = 82)	*t*/*z*/*χ*^2^	*p*-value
Man (%)	12 (44.44)	47 (57.32)	1.356	0.244
Age	47.44 ± 6.81	51.89 ± 7.98	−2.576	0.011
Hypertension (%)	18 (66.67)	36 (43.90)	4.211	0.040
Smoke (%)	8 (29.63)	27 (32.93)	0.101	0.750
Subarachnoid hemorrhage times (%)			4.566	0.033
<2	18 (66.67)	70 (85.37)		
≥2	9 (33.33)	12 (14.63)		
Aneurysm size (%)			4.375	0.224
≤5 mm	17 (62.96)	44 (53.66)		
5–15 mm	7 (25.93)	31 (37.80)		
15–20 mm	1 (3.70)	6 (7.32)		
≥20 mm	2 (7.41)	1 (1.22)		
Aneurysm location (%)			6.794	0.009
Anterior circulation aneurysm	19 (70.37)	34 (41.46)		
Other	8 (29.63)	48 (58.54)		
Modified Fisher grade (%)			12.377	0.006
I	5 (18.52)	31 (37.80)		
II	10 (37.04)	40 (48.78)		
III	9 (33.33)	9 (10.98)		
IV	3 (11.11)	2 (2.44)		
Hunt-Hess grade (%)			11.101	0.011
I	4 (14.81)	14 (17.07)		
II	14 (51.85)	61 (74.39)		
III	7 (25.93)	4 (4.88)		
IV	2 (7.41)	3 (3.66)		
Ca^+^ (mmol/L)	2.42 (2.31, 2.72)	2.51 (2.31, 2.71)	−0.330	0.741
Hcy (μmol/L)	22.18 ± 9.15	16.63 ± 7.08	3.239	0.002
PLT (10^9^/L)	229.70 ± 56.27	203.07 ± 54.56	2.162	0.033
RBC (10^12^/L)	4.97 ± 0.58	5.00 ± 0.50	−0.264	0.793
Hb (g/L)	144.85 ± 12.05	143.96 ± 15.40	0.271	0.787
HCT (%)	45.2 (42.3, 46.7)	45.0 (42.0, 47.8)	−0.337	0.736
WBC (10^9^/L)	11.3 (8.3, 14.5)	8.2 (6.2, 12.4)	−2.310	0.021
Glucose/potassium ratio	1.65 (1.46, 1.81)	1.29 (1.23, 1.36)	−3.327	0.001

Hcy, homocysteine; PLT, platelet; RBC, red blood cell; Hg, hemoglobin; HCT, hematocrit; WBC, white blood cell.

### Identifying the independent risk factors for CVS in aSAH patients after neurosurgical clipping

All the potential risk factors (*p* < 0.05) in the univariate regression analysis were included in the multivariate regression model. Multivariate logistic regression analysis revealed that age (OR = 0.843, 95% CI: 0.752–0.944, *p* = 0.003), aneurysm location (OR = 5.652, 95% CI: 1.030–31.016, *p* = 0.046), modified Fisher grade (*p* = 0.044), Hcy (OR = 1.170, 95% CI: 1.034–1.323, *p* = 0.013), WBC (OR = 1.202, 95% CI:1.015–1.425, *p* = 0.033), and glucose/potassium ratio (OR = 11.439, 95% CI: 1.253–104.393, *p* = 0.031) were independent risk factors for CVS in aSAH patients after neurosurgical clipping (Table 3).

**Table 3:** Univariate and multivariate logistic regression analyses of risk factors for CVS in the training set.

Variables	Univariate		Multivariate	
	OR (95% CI)	*p*-value	OR (95% CI)	*p*-value
Age	0.927 (0.873–0.985)	0.014	0.843 (0.752–0.944)	0.003
Hypertension	2.556 (1.027–6.357)	0.044		
**Subarachnoid hemorrhage times (%)**				
<2	Reference			
≥2	2.917 (1.065–7.989)	0.037		
**Aneurysm location (%)**				
Anterior circulation aneurysm	3.353 (1.316–8.546)	0.011	5.652 (1.030–31.016)	0.046
Other	Reference		Reference	
**Modified Fisher grade**				
I	Reference	0.012	Reference	0.044
II	1.550 (0.480–5.002)	0.463	17.903 (1.612–198.896)	0.019
III	6.200 (1.654–23.240)	0.007	29.161 (1.535–553.983)	0.025
IV	9.300 (1.230–70.333)	0.031	2.272 (0.021–246.934)	0.732
**Hunt-Hess grade**				
I	Reference	0.026		
II	0.803 (0.229–2.815)	0.732		
III	6.125 (1.169–32.100)	0.032		
IV	2.333 (0.284–19.172)	0.430		
Hcy (μmol/L)	1.089 (1.029–1.153)	0.003	1.170 (1.034–1.323)	0.013
PLT (10^9^/L)	1.009 (1.001–1.017)	0.036		
WBC (10^9^/L)	1.122 (1.011–1.244)	0.030	1.202 (1.015–1.425)	0.033
Glucose/potassium ratio	12.955 (2.871–58.467)	0.001	11.439 (1.253–104.393)	0.031

Hcy, homocysteine; PLT, platelet; WBC, white blood cell.

### The predictive nomogram development

**Figure 2:**
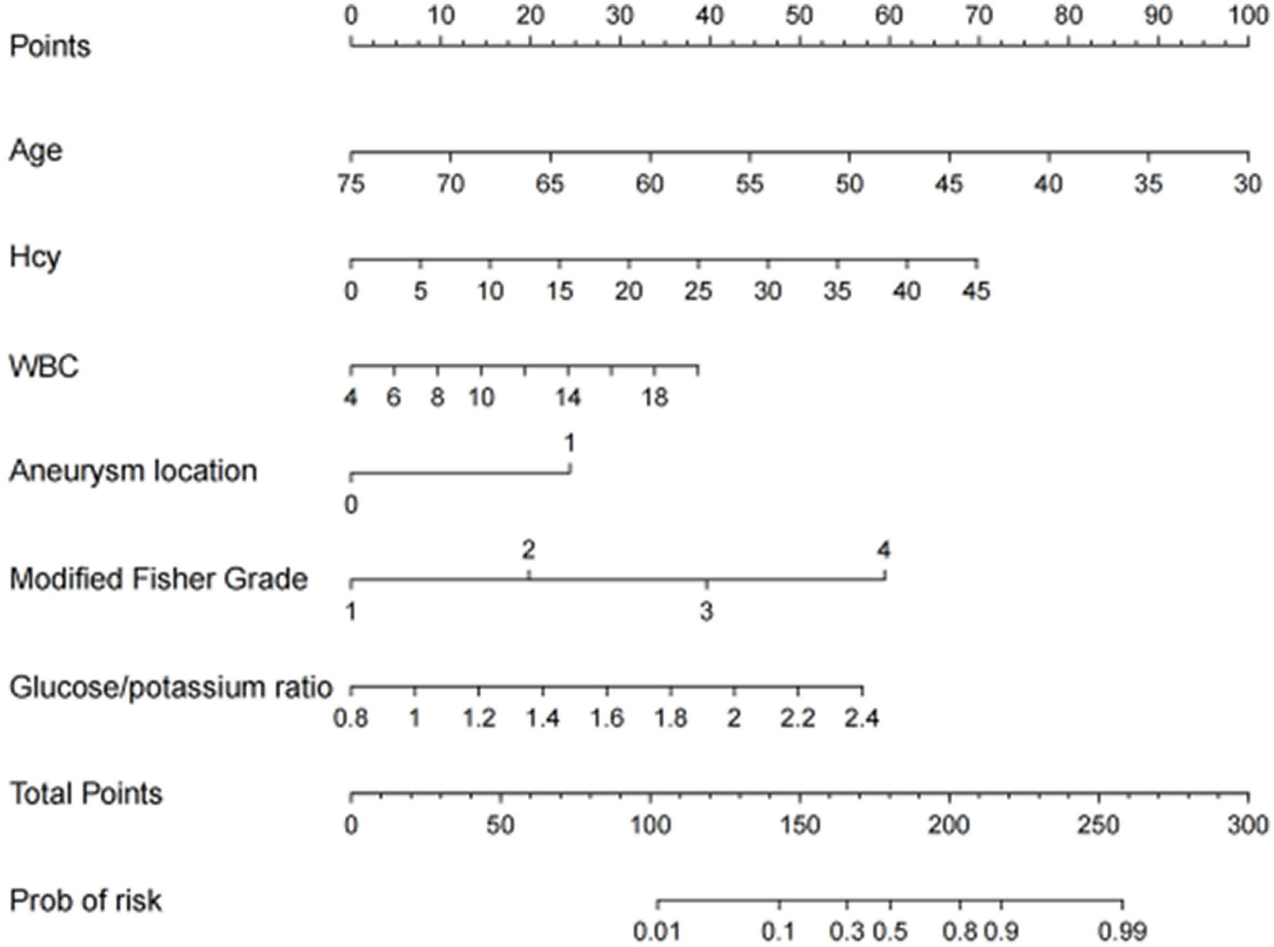
The nomogram for predicting the risk of CVS probability based on the 6 independent risk factors.

The nomogram was developed for predicting the risk of CVS in aSAH patients after neurosurgical clipping based on the results from the multivariate logistic model, which included six variables (Figure 2). A vertical line was drawn up to the “Point” axis to calculate the score of each variable, and the total score was summarized by the preliminary scores. The total score was located on the “Total Points” axis, and then, the predicted risk of CVS could be located on the bottom axis.

### The performance of the nomogram

**Figure 3:**
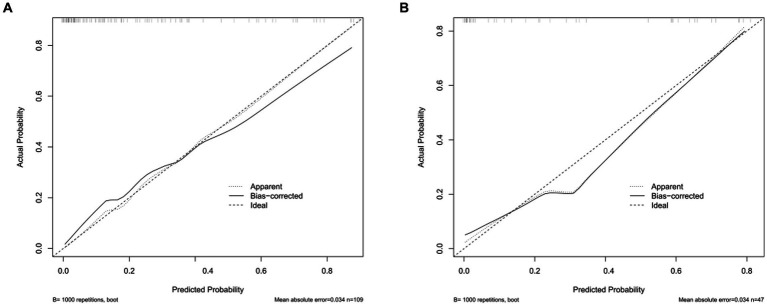
Calibration curves of the nomogram in the training set and the validation set. **(A)** The nomogram in the training set (*n* = 109). **(B)** The nomogram in the validation set (*n* = 47). The *y*-axis represents the observed rate of CVS, and the *x*-axis represents the nomogram-predicted probability of CVS. The dotted lines represented by the nomogram are closer to the diagonal gray lines representing a better prediction.

The calibration curve of the nomogram for the probability of CVS demonstrated a good agreement between prediction and observation for both sets (Figure 3). The Hosmer–Lemeshow *H* test indicated that the model did not depart from perfect fit, which had non-statistical significance in the training set (*p* = 0.3874) and validation set (*p* = 0.3154).

**Figure 4:**
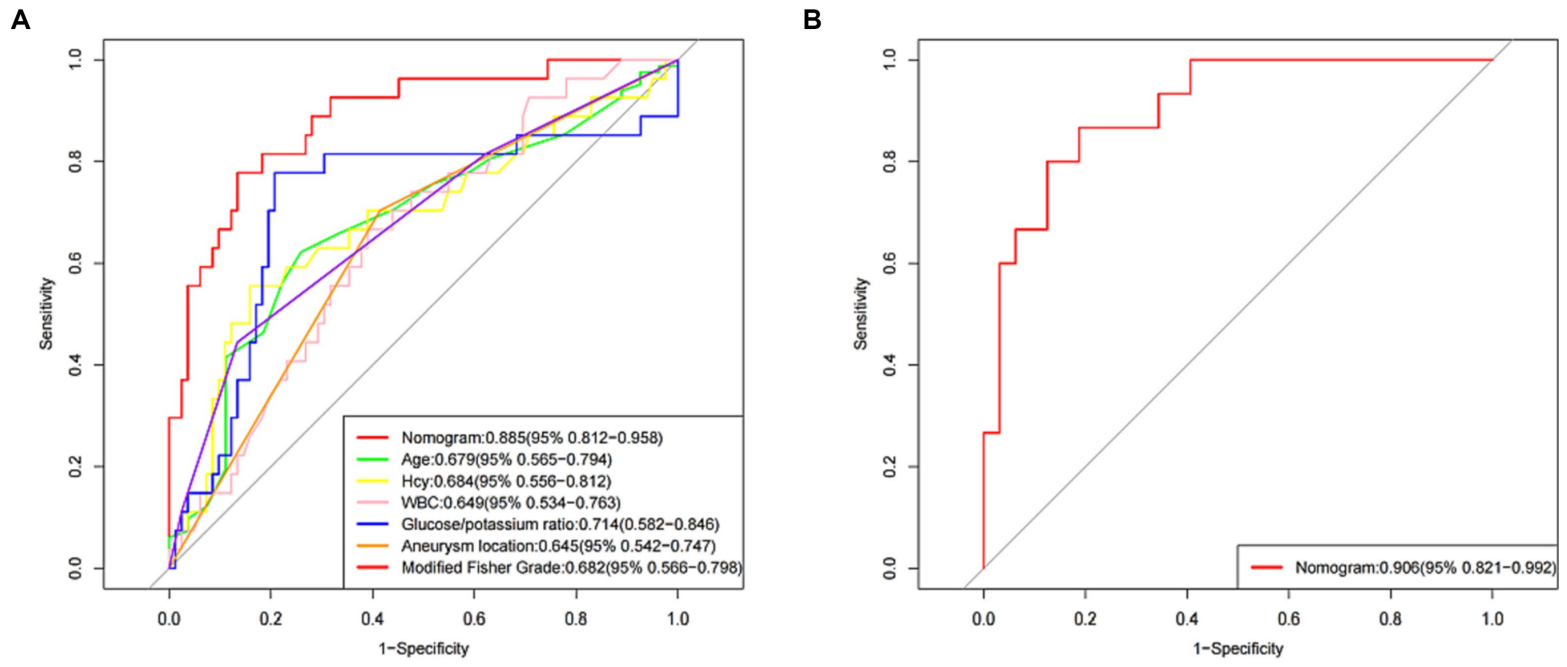
The receiver operating characteristic (ROC) curve of the nomogram in the training set and the validation set. **(A)** ROC in the training set. **(B)** ROC in the validation set.

The AUC for the nomogram was 0.885 (95% CI: 0.812–0.958) in the training set (Figure 4A) and was confirmed to be 0.906 (95% CI: 0.821–0.992) through internal validation in the validation set (Figure 4B), which demonstrated that the nomogram had a greater discriminatory performance. In addition, the discrimination ability of the nomogram calculated by the AUC was superior to the other risk factors in the training set: age (0.679, 95% CI: 0.565–0.794), Hcy (0.684, 95% CI: 0.556–0.812), WBC (0.649, 95% CI: 0.534–0.763), glucose/potassium ratio (0.714, 95% CI: 0.582–0.846), aneurysm location (0.645, 95% CI: 0.542–0.747) and modified Fisher grade (0.682, 95% CI: 0.566–0.798) (Figure 4A).

## Discussion

Aneurysm neurosurgical clipping is a common method for the clinical treatment of aSAH at present. It can effectively target areas that cannot be reached by conventional treatments, and the treatment’s effect is good, effectively reducing the clinical symptoms of patients. However, patients with aSAH are prone to experiencing CVS after clipping. Some studies suggested that craniotomy may exacerbated CVS, with influencing factors including the entry of blood clots and their metabolic by-products into the subarachnoid space, release of arachidonic acid and free radicals, and the spasmogenic response of cerebral arteries to surgical manipulation, etc. Furthermore, the resection and disruption of brain tissue could lead to the release of lipid peroxides such as arachidonic acid, resulting in increased synthesis of prostaglandins and free radicals (10, 13–15). Earlier research has indicated that these inflammatory substances could intensify CVS. Additionally, Woertgen’s study suggested that the occlusion of parent arteries with temporary clamping clips may be another factor exacerbating CVS after subarachnoid hemorrhage (16). From the surgical experience of paraclinoid aneurysms, Kazuo concluded that the number of temporary clamping clips and the frequency of temporary occlusion of the internal carotid artery were significantly correlated with CVS (17). All these findings implyed that mechanical stimulation of the vessel wall may be a crucial factor leading to or worsening CVS. CVS can result in the contraction of intracranial vascular smooth muscle and a temporary reduction in vascular diameter. As a result, transient ischemia occurs at the local blood supply site of the patient, leading to irreversible damage to the patient’s brain nerve tissue and significantly affecting the treatment outcome. Therefore, it is necessary to identify the factors that affect CVS in patients with aSAH after neurosurgical clipping.

The results of this study showed that 42 of 156 patients with aSAH who underwent clipping had CVS, with an incidence of 26.92%. Multivariate logistic regression analysis showed that age, Hcy levels, WBC count, the glucose/potassium ratio, aneurysm location, and the modified Fisher grade were independent risk factors for CVS.

The relationship between CVS and age remains controversial. Some studies have reported varying degrees of atherosclerosis in the vascular walls of elderly patients, resulting in a decline in cerebrovascular autoregulation. Consequently, elderly patients have been identified as a susceptible population for CVS. For instance, Lanzino et al. (18) analyzed data from 906 patients with aSAH admitted to 21 neurosurgical centers and found that CVS was more common in the older group. However, other studies suggested the opposite. Magge et al. (19) reported that age was related to the occurrence of CVS, and considered that young age was a high risk factor for CVS. Lazaridis and Naval (20) even suggested that patients younger than 35 years old were susceptible to CVS. The reason for this phenomenon may be that there was no hardening of the vascular wall in young patients, the vascular wall was sensitive to vasoconstrictor substances and the lumen was narrow and obvious. The results of our study suggested that relatively younger patients with aSAH were prone to CVS after surgery, and appropriate treatment plans and postoperative prevention measures should be specified for these patients.

High level Hcy can change the coagulation process, promote the aggregation of platelets, reduce the protective effect of high-density protein on blood vessels, and also mediate endothelial damage by releasing reactive oxygen species (21–23). CVS is based on the above reaction process of vascular endothelial cells, so high level of Hcy may indirectly promote the occurrence of CVS. In a study of 224 patients, McGirt et al. (24) identified WBC as a predictor of vasospasm. Other studies found that fever, WBC, and interleukin-6 levels in cerebrospinal fluid or serum were associated with vasospasm (25). Although the specific mechanism was not clear, it may be related to the fact that WBC can mediate and promote inflammatory response and hypercoagulable state, we believed that WBC can promote the development of CVS after clipping in patients with aSAH (26). The current hypothesis posited that CVS after SAH was regulated by nitric oxide (NO) (27). Huang et al. (28) pointed out that hyperglycemia exacerbated vasospasm after SAH and indicated that hyperglycemia may have an additional effect on cerebral CVS by regulating vascular function through NO and NO synthetase. Low serum potassium levels can cause vasoconstriction by inhibiting sodium/potassium pump in vascular smooth-muscle cells (29). In addition, potassium was involved in the production of NO, and the decreased plasma potassium level may affect the production of NO, which may impair vasodilatation (30). Consistent with previous studies, we found that the serum glucose/potassium ratio was a risk factor for CVS (31). Compared with simple blood glucose or serum potassium ion, glucose/potassium ratio as a more comprehensive index may have more reference significance for vascular effects. Based on the above, strengthening the monitoring and intervention of abnormal indicators in aSAH patients during the perioperative and postoperative period may be beneficial to avoid the occurrence of CVS.

Some studies showed an increased incidence of CVS in patients with anterior circulation aneurysms and the location of aneurysms was considered to be an independent risk factor for CVS (32, 33). Anterior circulation aneurysms, especially anterior cerebral artery aneurysms, anterior communicating artery aneurysms and middle cerebral artery aneurysms, had a higher incidence of CVS than posterior circulation aneurysms (34–36). The possible reason was that aneurysms in the anterior circulation were more likely to be stretched or even twisted during surgery. Early CVS may result from mechanical stimulation at the time of the intervention. Therefore, before the surgery for clipping aneurysms, we can provided sufficient operating space for the surgery and reduced traction and stimulation on the cerebral vessels through precise operations, dissecting the cerebral pool, slowly releasing cerebrospinal fluid, or releasing the arachnoid membrane on the surface of the blood vessels, which were particularly important during surgery for anterior cerebral circulation aneurysms.

Animal experiments and clinical studies confirmed that subarachnoid blood clots can cause CVS, and the location and severity of CVS were related to the location and size of subarachnoid blood clots (37). The modified Fisher grade proposed by Frontera et al. (12) was commonly used to speculate on the risk of CVS. The modified Fisher grade was significantly correlated with the occurrence of CVS, with higher grading being more prone to CVS. After the rupture and bleeding of an aneurysm, severe CVS was almost inevitable when initially CT showed thick blood accumulation, while CVS was rarely seen when there were no blood clots. Zaidat et al. (38) found that modified Fisher grade ≥III and Hunt-Hess grade ≥IV were independent risk factors for the occurrence of CVS. In preoperative patients with severe cerebral hemorrhage, having a high modified Fisher grade and Hunt-Hess grade suggests relatively insufficient compensatory ability for ischemia, making them more susceptible to CVS. In our study, Hunt-Hess grade did not emerge as an independent risk factor, possibly due to sample size limitations, which will require further research in the future. Therefore, if circumstances allow, conservative treatment can be considered before craniotomy, including promoting more rest and providing a light, easily digestible diet to potentially improve or lower the modified Fisher grade in patients. This approach may reduce the occurrence of CVS after surgery.

### Limitations

We need to acknowledge some limitations of this study. First, it was a single-center observational study, lacking external validation. Secondly, the sample size and unforeseen confounding factors could potentially influence the experimental results. And we did not perform a more detailed classification for factors such as hypertension, smoking, and aneurysm location. Finally, we only included clinical data from patients at admission. However, repeated measures of clinical data may provide more accurate insights into predicting CVS in patients with aSAH after neurosurgical clipping.

## Conclusion

In summary, this study found that the occurrence of CVS in patients with aSAH after neurosurgical clipping was related to age, Hcy levels, WBC count, the glucose/potassium ratio, aneurysm location, and the modified Fisher grade. The nomogram constructed in this study performed well in predicting CVS in aSAH patients after neurosurgical clipping. Based on these findings, targeted plans can be proposed in clinical practice to enhance comprehensive detection and care during the perioperative period and after surgery, with the aim of preventing the occurrence of CVS.

## Data availability statement

The original contributions presented in the study are included in the article/supplementary material, further inquiries can be directed to the corresponding author.

## Ethics statement

This study has been reviewed and approved by the Medical Ethics Committee of Gansu Provincial Hospital, 2023-587. Written informed consent for participation was not required from the participants or the participants’ legal guardians/next of kin in accordance with the national legislation and institutional requirements.

## Author contributions

YZ: Conceptualization, Data curation, Formal analysis, Investigation, Methodology, Project administration, Resources, Software, Supervision, Validation, Visualization, Writing – original draft, Writing – review & editing. DQ: Conceptualization, Data curation, Formal analysis, Investigation, Methodology, Project administration, Software, Validation, Writing – review & editing. YM: Conceptualization, Data curation, Formal analysis, Methodology, Writing – review & editing. BL: Conceptualization, Data curation, Formal analysis, Investigation, Software, Supervision, Writing – review & editing. ZZ: Conceptualization, Data curation, Formal analysis, Investigation, Methodology, Project administration, Validation, Writing – review & editing. EC: Funding acquisition, Investigation, Project administration, Supervision, Validation, Writing – original draft, Writing – review & editing.
